# Ganglioside GD2 in reception and transduction of cell death signal in tumor cells

**DOI:** 10.1186/1471-2407-14-295

**Published:** 2014-04-28

**Authors:** Igor I Doronin, Polina A Vishnyakova, Irina V Kholodenko, Eugene D Ponomarev, Dmitry Y Ryazantsev, Irina M Molotkovskaya, Roman V Kholodenko

**Affiliations:** 1 Shemyakin-Ovchinnikov Institute of Bioorganic Chemistry, Russian Academy of Sciences, Miklukho-Maklaya St., 16/10, Moscow 117997, Russia; 2 Orekhovich Institute of Biomedical Chemistry, Russian Academy of Medical Sciences, 10, Pogodinskaya St., Moscow 119121, Russia; 3 School of Biomedical Sciences, Faculty of Medicine, The Chinese University of Hong Kong, Shatin NT, Hong Kong, China

**Keywords:** GD2, Anti-GD2 mAbs, Cytotoxicity, Cell death, Tumor-associated gangliosides

## Abstract

**Background:**

Ganglioside GD2 is expressed on plasma membranes of various types of malignant cells. One of the most promising approaches for cancer immunotherapy is the treatment with monoclonal antibodies recognizing tumor-associated markers such as ganglioside GD2. It is considered that major mechanisms of anticancer activity of anti-GD2 antibodies are complement-dependent cytotoxicity and/or antibody-mediated cellular cytotoxicity. At the same time, several studies suggested that anti-GD2 antibodies are capable of direct induction of cell death of number of tumor cell lines, but it has not been investigated in details. In this study we investigated the functional role of ganglioside GD2 in the induction of cell death of multiple tumor cell lines by using GD2-specific monoclonal antibodies.

**Methods:**

Expression of GD2 on different tumor cell lines was analyzed by flow cytometry using anti-GD2 antibodies. By using HPTLC followed by densitometric analysis we measured the amount of ganglioside GD2 in total ganglioside fractions isolated from tumor cell lines. An MTT assay was performed to assess viability of GD2-positive and -negative tumor cell lines treated with anti-GD2 mAbs. Cross-reactivity of anti-GD2 mAbs with other gangliosides or other surface molecules was investigated by ELISA and flow cytometry. Inhibition of GD2 expression was achieved by using of inhibitor for ganglioside synthesis PDMP and/or siRNA for GM2/GD2 and GD3 synthases.

**Results:**

Anti-GD2 mAbs effectively induced non-classical cell death that combined features of both apoptosis and necrosis in GD2-positive tumor cells and did not affect GD2-negative tumors. Anti-GD2 mAbs directly induced cell death, which included alteration of mitochondrial membrane potential, induction of apoptotic volume decrease and cell membrane permeability. This cytotoxic effect was mediated exclusively by specific binding of anti-GD2 antibodies with ganglioside GD2 but not with other molecules. Moreover, the level of GD2 expression correlated with susceptibility of tumor cell lines to cytotoxic effect of anti-GD2 antibodies.

**Conclusions:**

Results of this study demonstrate that anti-GD2 antibodies not only passively bind to the surface of tumor cells but also directly induce rapid cell death after the incubation with GD2-positive tumor cells. These results suggest a new role of GD2 as a receptor that actively transduces death signal in malignant cells.

## Background

Tumor-associated gangliosides are very promising target molecules for the development of new anti-cancer drugs. Gangliosides are glycosilated lipid molecules belonging to the class of glycosphingolipids and containing the sialic acid residues in their carbohydrate structure. Quite a few gangliosides including GD2, GM2, GD3, NGcGM3 and OAcGD2 are expressed at very high levels on the plasma membrane of several tumor cells of neuroectodermal origin (such as neuroblastomas, melanomas, gliomas), as well as on the cells of small cell lung cancers and lymphomas.

As a potential target molecule for anti-tumor therapy, ganglioside GD2 has certain advantages when compared to other tumor-associated gangliosides since this glycolipid is highly expressed in tumor cells and it is not expressed at all, or expressed at a very low level in normal cells. Specifically, in normal non-malignant tissues, GD2 expression is mostly restricted to neurons, skin melanocytes and peripheral nerves. Moreover, on the surface of normal cells, GD2 is a minor ganglioside, comprising 1-2% of total amount of gangliosides, and its level of expression is 3-8-fold lower in comparison with other tumor-associated gangliosides such as GD3 [1]. In tumors the highest level of GD2 expression is observed on the cell surface of almost all types of the primary neuroblastomas reaching ~10^7^ molecules per cell [2,3]. In addition, GD2 is detected in about 75% of primary and metastatic melanomas [4]. GD2 is also expressed in variety of other tumors including bone and soft-tissue sarcomas, small cell lung cancer, and brain tumors [5,6].

Today, one of the most promising approaches for cancer immunotherapy is the treatment of cancer patients with monoclonal antibodies (mAbs) directed against tumor-associated molecules including ganglioside GD2. Several monoclonal antibodies specific for the GD2 were recently used in clinical trials [7]. The anti-GD2 mAbs appear to act mainly through binding to the cell surface of tumor cells and activation of complement system that leads to complement-dependent lysis and/or antibody-mediated cellular cytotoxicity that involve immune cells as effectors [8]. At the same time, several studies suggested that anti-GD2 mAbs may cause direct induction of cell death in a number of tumor cell lines [9-11]. However it has not been thoroughly investigated. The functional role of GD2 ganglioside in this process has not been demonstrated, and possibility of cross-reactivity of anti-GD2 mAbs with other gangliosides and glycosylated proteins was not yet tested.

In this study we demonstrated a new role of ganglioside GD2 as a receptor for induction of non-classical cell death of GD2-positive tumor cells of various origins. We found that anti-GD2 antibodies specifically interacted with GD2 resulting in direct induction of mitochondria-dependent cell death. We also found that the level of GD2 expression directly correlated with susceptibility of these cells to cytotoxicity induced by anti-GD2 antibodies. Thus, our study establishes a new role of GD2 as a functionally active biomarker for anti-cancer therapy.

## Methods

### Cell lines and hybridomas

EL-4 (mouse lymphoma), L1210 (mouse lymphoma), Jurkat (human lymphoma) cell lines were cultured in RPMI-1640; IMR-32 (human neuroblastoma) and Neuro-2A (mouse neuroblastoma) cell lines were cultured in EMEM medium; human melanomas mS and A375 were cultured in DMEM medium. All culture mediums were supplemented with 10% heat-inactivated fetal bovine serum (FBS, HyClone), 2 mM *L*-glutamine and antibiotic/antimycotic solution (Sigma). Hybridoma cells HB9326 were maintained in Hybri-Max RPMI-1640 medium, supplemented with 10% FBS, 2 mM *L*-glutamine and antibiotic/antimycotic solution. All cell lines except mS were kindly provided by Dr. E.V. Svirshchevskaya (Shemyakin-Ovchinnikov Institute of Bioorganic Chemistry), cell line mS was kindly provided by Dr. S.E. Dmitriev (Belozersky Institute of Physico-Chemical Biology, Lomonosov Moscow State University), HB9326 hybridoma cell line was originally purchased from the American Type Culture Collection (ATCC) and kindly provided by Dr. Telford (Experimental Transplantation and Immunology Branch, NCI, National Institutes of Health).

### Antibodies and reagents

Mouse ME361 (S2A) antibody produced by HB9326 hybridoma cells were purified as described previously [12]. GD2-specific antibodies ME361 were purified from mouse ascites by affinity chromatography. Other anti-GD2 14G2a mAbs were purchased from Millipore Inc. Anti-GM2/GD2 synthase and anti-ALCAM antibodies, siRNA and primers for GM2/GD2 and GD3 synthases were purchased from Santa Cruz Biotechnology Inc.

### Flow cytometry

Staining of EL-4, L1210, Jurkat, IMR-32, Neuro-2A, mS, and A375 cells with two type of GD2-specific antibodies 14G2a and ME361 was performed as described previously [11]. In brief, cells were detached from the culture plates (adherent cells were trypsinized and washed two times with PBS) and were incubated with AlexaFluor-488-labeled or unlabeled anti-GD2 mAbs (1 μg per 10^6^ cells) for 1 h and then washed in PBS supplemented with 1% FBS and 0.02% sodium azide. After that, in the case of unlabeled anti-GD2 mAbs, the cells were incubated with FITC-labeled anti-mouse IgG (1:1000) for 40 min, and then twice washed in PBS. All procedures were performed at 4°C. The samples were immediately analyzed using EPICS Coulter XL-MCL flow cytometer. In each sample at least 5,000 events were collected. For all samples, the analysis was performed in triplicate. The data was analyzed using FlowJo and WinMDI software.

### Microscopy and immunofluorescence

EL-4, IMR-32 and mS cell lines were grown on glass coverslips (Fisher Scientific) placed into 6-well tissue culture plates (Greiner). The cells that were grown to 80% confluence were subsequently washed with PBS and fixed with 2% paraformaldehyde (PF) for 30 min at room temperature (RT). After which, cells were washed twice with PBS and quenched with 50 mM NH_4_Cl for 10 min. After washing with PBS, the cells were blocked with PBS containing 10% FBS and incubated with 100 μl anti-GD2 mAbs (10 μg/ml) for 1 h at 4°C and then with FITC-labeled anti-mouse IgG (titer 1:1000) for 40 min at 4°C. Stained cells were fixed with 2% PF for 30 min at RT, and then sequentially washed in PBS and distilled water. Counterstaining was performed with Hoechst 33342 (0.5 μg/ml) for 10 min, and finally cell preparations were mounted in Mowiol (Calbiochem-Behring GmbH). Slides were analyzed using a confocal laser scanning microscope EZ-C1 Eclipse TE2000 (Nicon) equipped with a Plan Apo 40X and 60X objectives. Images were collected with EZ-C1 program and processed with EC1 Viewer (Nikon).

### Ganglioside purification and quantitation

Total cellular gangliosides were extracted from GD2-positive (EL-4, mS, IMR-32) and GD2-negative (Jurkat, L1210, A375, Neuro-2A) cell lines. Total lipid extracts were obtained by multiple extractions of the lyophilized cell pellets (5 × 10^7^ cells) with chloroform/methanol (2:1 and 1:2 (v/v) at 4°C. At each stage, the hydrophobic extracts were separated from the pellet by centrifugation (12000 g, 10 min). Total lipid extracts were washed with water five times to separate gangliosides as described by Folch *et al*. [13]. Gangliosides in the aqueous phase were further purified on the cartridge Strata-X (33 μm, 60 mg/3 ml; Phenomenex) and their concentrations were assessed by the modified resorcinol method [14]. High-performance thin layer chromatography (HPTLC) analysis of gangliosides was performed on silica gel using 60 HPTLC plates (Merck) in chloroform/methanol/0.2% aq. CaCl_2_ (60:40:9, v/v/v) system. Then plates were dried in the flow of cool air, incubated at 110°C for 15 s, and visualized by spraying with resorcinol-HCl reagent and further heating for 20 min at 110°C. Total cellular ganglioside content was determined as the sum of individual gangliosides measured by HPTLC densitometry (Shimadzu CS-920) using known concentrations of bovine liver GM1 (0.1 – 1 μg) as standard.

### Viability and cell death assays

#### Propidium Iodide (PI) assay

Analyses of cell death as determined by DNA fragmentation were performed using propidium iodide (PI) staining in accordance to previously described method [15] with modifications [16]. The tumor cells (5 × 10^5^ cells per sample) were incubated with anti-GD2 mAbs at concentration of 5 μg/ml for 24 h under standard culture conditions. After incubation the cells were fixed and permeabilized with ice cold ethanol at 4°C for 60 min, and washed twice with PBS by centrifugation for 10 min at 300 g. The cell pellets were resuspended in DNA staining buffer (PBS, 20 μg/ml PI (Sigma), 20 μg/ml RNase A (Fermentas)), and further incubated for 30 min at RT. For all samples, cell death analysis was performed in triplicate. An EPICS Coulter XL-MCL flow cytometer was used to evaluate percent of cells with lower intensity of fluorescence in FL3 channel, which is characteristic of cells with fragmented DNA. In each sample at least 5,000 events were registered. Data processing was performed using FlowJo and WinMDI software.

#### MTT assay

Antibody-induced decrease in cell viability was analyzed by colorimetric MTT (3-[[4,5]-dimethylthiazol-2-yl]-2,5-diphenyltetrazolium bromide; purchased from Sigma) assay previously described by Denizot and Lang [17]. Briefly, tumor cells were cultured in 96-well flat-bottomed tissue culture plates (10^4^ cells/well, Greiner) with serial dilutions of mAbs ME361 and 14G2a (concentration range was from 0.031 to 10.000 μg/ml) for 72 h under standard culture conditions. After incubation, the MTT solution (250 μg/ml final concentration) was added to each sample for 4 h. The optical density (OD) was read in a Multiscan FC microplate reader (Thermo Scientific) at a test wavelength of 540 nm. Cell viability was measured as ratio of OD_540_ of cells treatment with anti-GD2 mAbs to OD_540_ of control cells. All MTT experiments for each cell line were reproduced at least three times.

### Apoptotic volume decrease (AVD)

Apoptotic volume decrease of EL-4 cells was detected by flow cytometry. Intact untreated cells or cells treated with anti-GD2 antibodies were distinguished as normal and shrunken populations by the changes in forward and side light scatter (FCS/SSC) characteristics. Cells with apoptotic volume decrease had reduced mean of forward scatter and increased mean of side scatter as compared with normal cells. In each sample at least 5,000 events were registered. The data was analyzed using FlowJo and WinMDI software.

### Caspase-3 activation assay

Evaluation of caspase-3 activation was performed in accordance with the method described earlier [18]. 2 × 10^6^ of untreated or treated with anti-GD2 mAbs EL-4 cells were washed once with PBS. Then, the cell lysate was prepared using RIPA-buffer. 20 μl of the lysate was placed in each well of a 96-well plate and the volume was adjusted to 200 μl buffer (100 mM HEPES, 20% glycerol, 5 mM DTT, 0.5 mM EDTA). The plate was incubated for 30 min at +37°C and then solution of fluorescently labeled caspase substrate Z-DEVD-AFC (10 μM) was added to each well. The fluorescence intensity was measured using Glomax spectrofluorometer (Promega, USA) at wavelengths on excitation and emission 400 nm and 505 nm, respectively.

### Plasma membrane permeability assay

The loss of plasma membrane integrity was analyzed using of fluorescent DNA binding dye 7-AAD (7-aminoactinomycin D; purchased from Sigma). EL-4 cells were washed once in PBS and resuspended in 0.5 ml of staining solution (PBS with 2 μg/ml 7-AAD). 7-AAD fluorescence of cells was analyzed by flow cytometry using FL3-channel. In each sample at least 5,000 events were collected. The data was analyzed using FlowJo and WinMDI software.

### Assessment of mitochondrial membrane potential in living cells

Mitochondrial membrane potential (ΔΨm) was measured using fluorescent dye 3,3′-dihexyloxacarbocyanine iodide (DiOC_6_(3)) and 5,5′,6,6′-tetrachloro-1,1′,3,3′-tetraethylbenzimi-dazolylcarbocyanine iodide (JC-1) (Sigma). The cell suspension was adjusted to a density of 1 × 10^6^ cell/ml and incubated in complete medium for 15 min at RT in the dark with 20 nM DiOC_6_(3) or with 2 μg/ml JC-1. After which, the cells were washed twice in cold PBS, suspended in a total volume of 500 μl and analyzed by flow cytometry (FL1-channel for DiOC_6_(3), or FL1 and FL2 channels for JC-1). In each sample at least 5,000events were collected. The data was analyzed using FlowJo and WinMDI software.

### ELISA

Polystyrene microtiter plates (Greiner) were coated with gangliosides GD2, GM2, GD1b and GD3 that were obtained according to the method applied in our previous work [19], or kindly provided by Dr. Mikhalyov (Institute of Bioorganic Chemistry, Russia Academy of Sciences) at concentration 0.25 μg in 100 μl of 70% methanol per well. Following air drying, all wells of the plate were blocked with 2% BSA in PBS-T (0.05% Tween 20 in PBS) in 100 μl per well for 2 h at RT. Antibodies (100 μl per well in PBS-T) were added in triplicates at different concentrations. Following incubation for 2 h at 37°C and washing with PBS-T, HRP-goat anti-mouse IgG (1:12000) were added. After incubation for 1 h at 37°C and further washing, TMB color reaction was performed and OD was read using Multiscan FC microplate reader (Thermo Scientific) at 450 nm. Percent of cross-reactivity was measured as ratio of OD_450_ of TMB substrate in GM2-, GD1b- or GD3-coated wells to OD_450_ of TMB substrate in GD2-coated wells.

The amount of gangliosides adsorbed to each well was determined by using fluorescent-labeled gangliosides BODIPY-FL-C5-GM1 and BODIPY-FL-C5-GD3 (kindly provided by Dr. Mikhalyov). Fluorescent probes were coated at the same concentration as unlabeled gangliosides (0.25 μg in 100 μl per well in 70% methanol), and the same operations were performed for fluorescent probes except adding of antibodies. At the last stage BODIPY-labeled gangliosides GM1 and GD3 that were adsorbed on surfaces of the wells were subsequently dissolved in methanol and fluorescence was measured using a Dynatech Micro FLUOR Reader (excitation 490 nm, emission 510–570 nm). The amount of gangliosides that were adsorbed on the wells was measured using proper calibration curve (linear regression: RFU BODIPY-FL-C5-GD3 = 20.726 + (271.329 × amount of ganglioside per well), RFU BODIPY-FL-C5-GM1 = 36.396 + (248.714 × amount of ganglioside per well, RFU – relative fluorescence units). All experiments were repeated three times.

### Modulation of GD2 expression

#### Downregulation of GD2 expression using PDMP inhibitor

In the initial experiments we determined optimal concentration of PDMP inhibitor and time of incubation to downregulate GD2 expression in EL-4 cells. EL-4 cells were treated with different concentrations of PDMP (at rage of 5–50 μM) for 2–7 days. The expression of GD2, cell viability, and cell death were analyzed by flow cytometry using surface staining for GD2, PI-, and MTT-tests. In these experiments, the cells were treated with 2.5-100 μM PDMP and incubated for 72 h. After selection of optimal concentration, EL-4 cells were cultured for 6 days in the presence of 15 μM PDMP before the analysis of cytotoxicity induced by treatment with anti-GD2 antibodies.

#### Knockdown of GM2/GD2 and GD3 synthases by siRNA

siRNA for mouse GM2/GD2 or GD3 synthases were purchased from Santa Cruz Inc. The cells were transfected with these siRNAs using lipophilic agent Lipofectamine-2000 (Invitrogen) according to the manufacturer’s instructions. Cells were harvested 48 h post-transfection and further incubated with anti-GD2 mAbs for 24 h followed by performing PI-test.

### Western blot analysis

Protein lysates of EL-4 cells were prepared using RIPA buffer (Assay Design). The proteins from cell lysate were fractionated in SDS-PAGE, and were transferred onto nitrocellulose membranes using a semi**-**dry transfer device V10-SDB (Biostep). Membranes were further incubated in blocking buffer (0.05% Tween 20, 5% nonfat dried milk in PBS) for 1 h at RT, followed by incubation in primary anti-GM2/GD2 synthase antibody (10 μg/ml) for 1 h at RT in PBS supplemented with 0.05% Tween 20 (PBS-T). After washing several times with PBS-T, the membranes were incubated for 1 h in HRP-conjugated secondary antibody (diluted 1:2000) at RT, and then were washed four times with PBS-T. The immunoreactive proteins were visualized using the Metal Enchanced DAB Substrate Kit (Thermo Scientific) according to the manufacturer’s instructions.

### RNA isolation and cDNA synthesis

Cells transfected with siRNA that target GM2/GD2 synthase or control cells were dissolved in 0.5 ml of Trizol reagent for isolation of the total RNA as described by the manufacturer (Invitrogen). All RNA extractions were carried out in a chemical hood using RNAse-free labware. RNA quality and quantity were evaluated by agarose gel electrophoresis and UV spectrometry (NanoVue, GE Helthscare). Samples were stored at −80°C until used. For reverse transcription reaction, 2 μg of total RNA was reversely transcribed using MMLV-RT kits according to the manufacturer’s protocol (Evrogen).

### Real time RT-PCR

A ten-fold serial dilution of the cDNA derived from EL-4 cells was prepared in order to make standard curves and determination of PCR efficiency primers for the GM2/GD2 synthase gene (Santa Cruz Biotechnology) and GAPDH housekeeping gene (Evrogen). For performance of real-time RT-PCR we used a DT-96 PCR machine (DNA-Technology LLC), and each reaction was performed in a total volume of 20 μl containing 2 μl of cDNA of the test sample or control sample (standard curve) with 5xSybrGreen-mix prepared according to the manufacturer's protocol (Eurogen). Final concentrations of the primer sets and MgCl_2_ were 10 μM and 3 mM, respectively. After the denaturation step at 95°C for 5 min, the amplification program was set at 40 cycles each consisting of denaturation at 95°C for 15 s followed by annealing at 58°C for 10s, extension at 72°C for 3 min, followed by detection at the specified acquisition temperature. Melting curve analysis was used for amplicon`s size estimation. Negative controls, samples without reverse transcription or cDNA template were included with every PCR run and were always negative (not shown). Relative gene expression was determined as the ratio of the GM2/GD2 synthase gene to the internal reference gene expression (GAPDH) based on the Ct values using QGENE software.

### Statistical analysis

Graphs were created using SigmaPlot and MS Excel software. These results were presented as Mean ± S.E. of at least three independent experiments, or one representative experiment of three was shown. Statistical analysis was performed using Student's t-test, Mann–Whitney Rank Sum Test, Analysis of Variance (ANOVA), whereas differences between means were inspected with Dunnett’s multiple comparison and Student-Newman-Keuls multiple comparison post-hoc tests. Significance levels of P < 0.05 were considered statistically reliable.

## Results

### Selection of relevant GD2-positive and GD2-negative tumor cell lines

**Figure 1:**
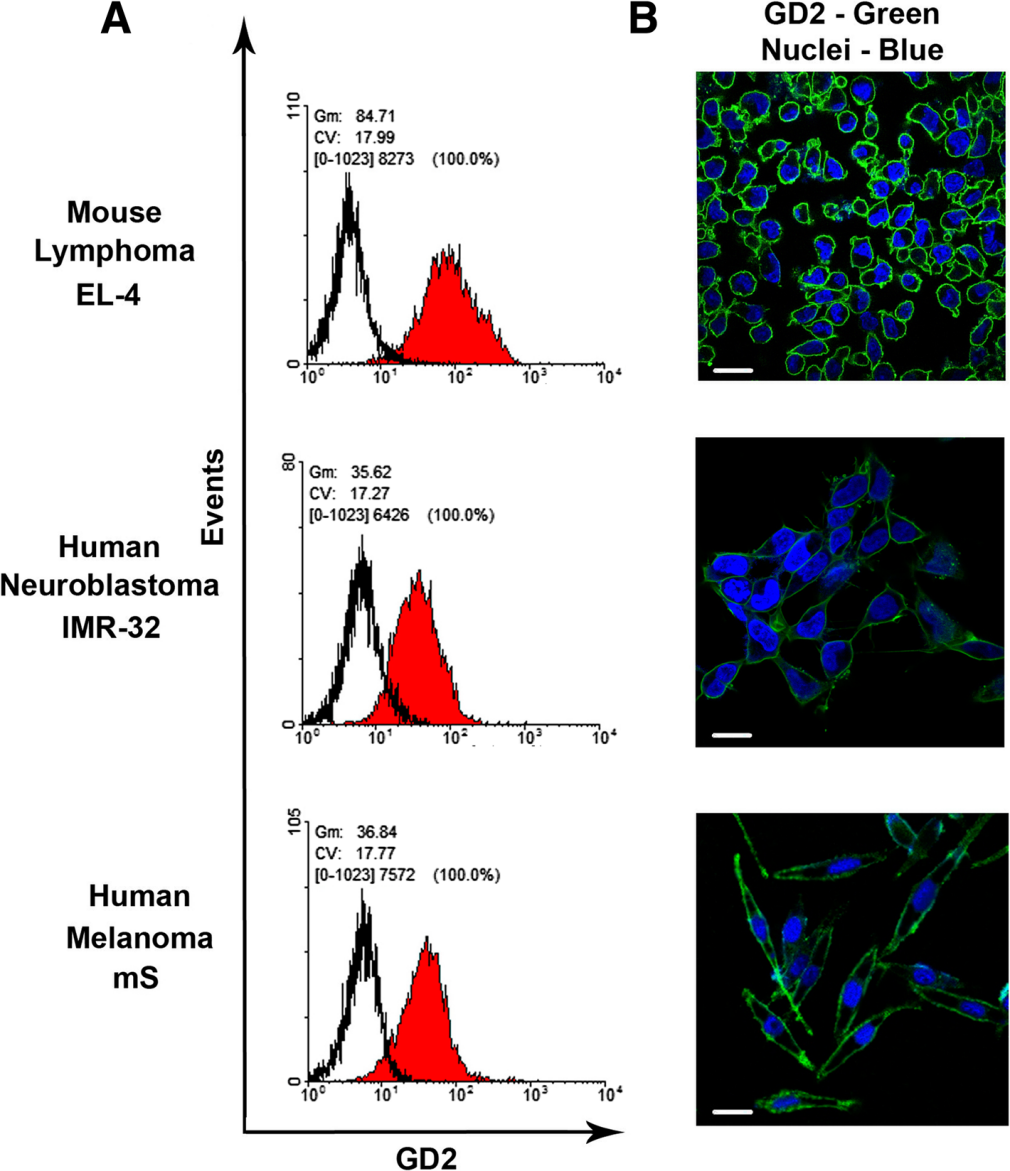
**Expression of GD2 on the cell surface of EL-4, IMR-32, and mS tumor cell lines.** Flow cytometry analysis of the cells stained with anti-GD2 antibodies conjugated with AlexaFluor488 (14G2a antibodies; 5 μg/ml; see *Methods*) is shown in **(A)**. Filled histograms (red color) show staining with anti-GD2 mAbs, empty histograms – staining with an isotype control. Confocal imaging of EL-4, IMR-32, and mS cells stained with anti-GD2 conjugated with AlexaFluor488 (14G2a antibodies; 5 μg/ml; see *Methods*) is shown in **(B)**. The staining with anti-GD2 mAb is shown in green color; the nuclei were counterstained with Hoechst 33342 (shown in blue). Bar scale: 50 μm.

We have analyzed the expression of ganglioside GD2 on various tumor cell lines of different origin by performing surface staining of the cells with anti-GD2 mAbs (not shown). Based on these data, we selected three cell lines with the highest expression level of GD2: mouse lymphoma EL-4, human neuroblastoma IMR-32, and human melanoma mS; and three cell lines either without, or with very low levels of GD2 expression: human Jurkat lymphoma, mouse neuroblastoma Neuro-2A, human melanoma A375. We have performed a surface staining of the cells with anti-GD2 mAb 14G2a directly conjugated with AlexaFluor488 and analyzed expression of GD2 by flow cytometry. All three selected GD2-positive cell lines were characterized by a high and uniform expression of ganglioside GD2 on the cell surface (Figure 1), while GD2-negative cell lines did not express GD2 as determined by flow cytometry (Additional file 1).

The representative histogram shown in Figure 1A demonstrates an increase in mean fluorescence intensity (MFI) of GD2 expression as determined by staining of the cells with the anti-GD2 antibodies 14G2a when compared to proper isotype control antibodies. These results indicate that examined cell lines expressed GD2. However, there was variability in MFI levels of GD2 expression among cell lines of different origin. The MFI for GD2 on lymphoma EL-4 cells was 2.5 ± 0.3 fold higher than that of melanoma mS cells, and was 2.7 ± 0.4 fold higher for neuroblastoma IMR-32 cells. Immunofluorescence microscopy analysis showed a uniform expression of GD2 on the surface of all three examined GD2-positive tumor cell lines (Figure 1B). The similar results were obtained when cells were staining with other type of anti-GD2 mAb ME361 (not shown). Thus, we have shown that the selected cell lines of different origin were GD2-positive. All of these cell lines were characterized by high expression level of ganglioside GD2 with the highest expression level in EL-4 lymphoma cells. Flow cytometry and immunofluorescence microscopy analysis of GD2-negative cell lines confirmed that ganglioside GD2 was not expressed in these cell lines. (Additional file 1A and B).

### Quantitative analysis of the total ganglioside and ganglioside GD2 expression in the chosen GD2 positive and GD2 negative cell lines

**Figure 2:**
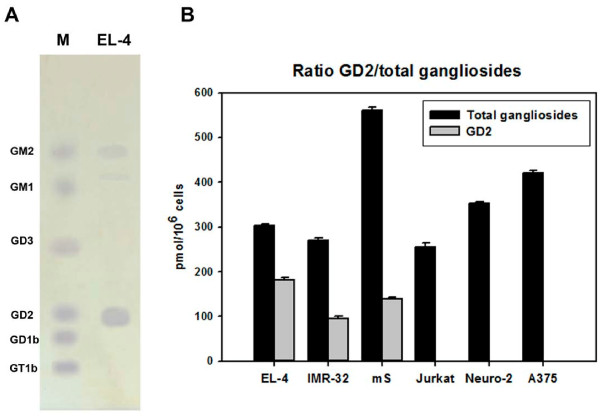
**Quantitative analysis of the total ganglioside content and proportion of ganglioside GD2.** HPTLC analysis of individual gangliosides in EL-4 cells was performed as described in *Methods and shown in ***(A)**. Ratio of ganglioside GD2 to the total amount of gangliosides in the different tumor cell lines is shown in **(B)**. The total cellular ganglioside content was determined as the sum of individual gangliosides measured by HPTLC densitometry.

To determine the proportion of ganglioside GD2 content to the total ganglioside amount, densitometric analysis was performed for ganglioside fractions isolated by HPTLC from selected cell lines. As seen in Figure 2A, the major gangliosides for EL-4 cells were GD2 and GM2. The percentages of amount of ganglioside GD2 of all gangliosides isolated from cell lines EL-4, IMR-32 and mS were 60%, 45% and 35%, respectively (Figure 2B). Ganglioside GD2 was not detected in ganglioside extracts of Jurkat, Neuro-2A and A375 cell lines.

Thus, we confirmed biochemically that we chose appropriate GD2-positive and GD2-negative cell lines to study physiological effects of anti-GD2 mAbs.

### Cytotoxic effects of two types of anti-GD2 mAbs 14G2a and ME361 on GD2-positive and GD2-negative tumor cell lines

**Figure 3:**
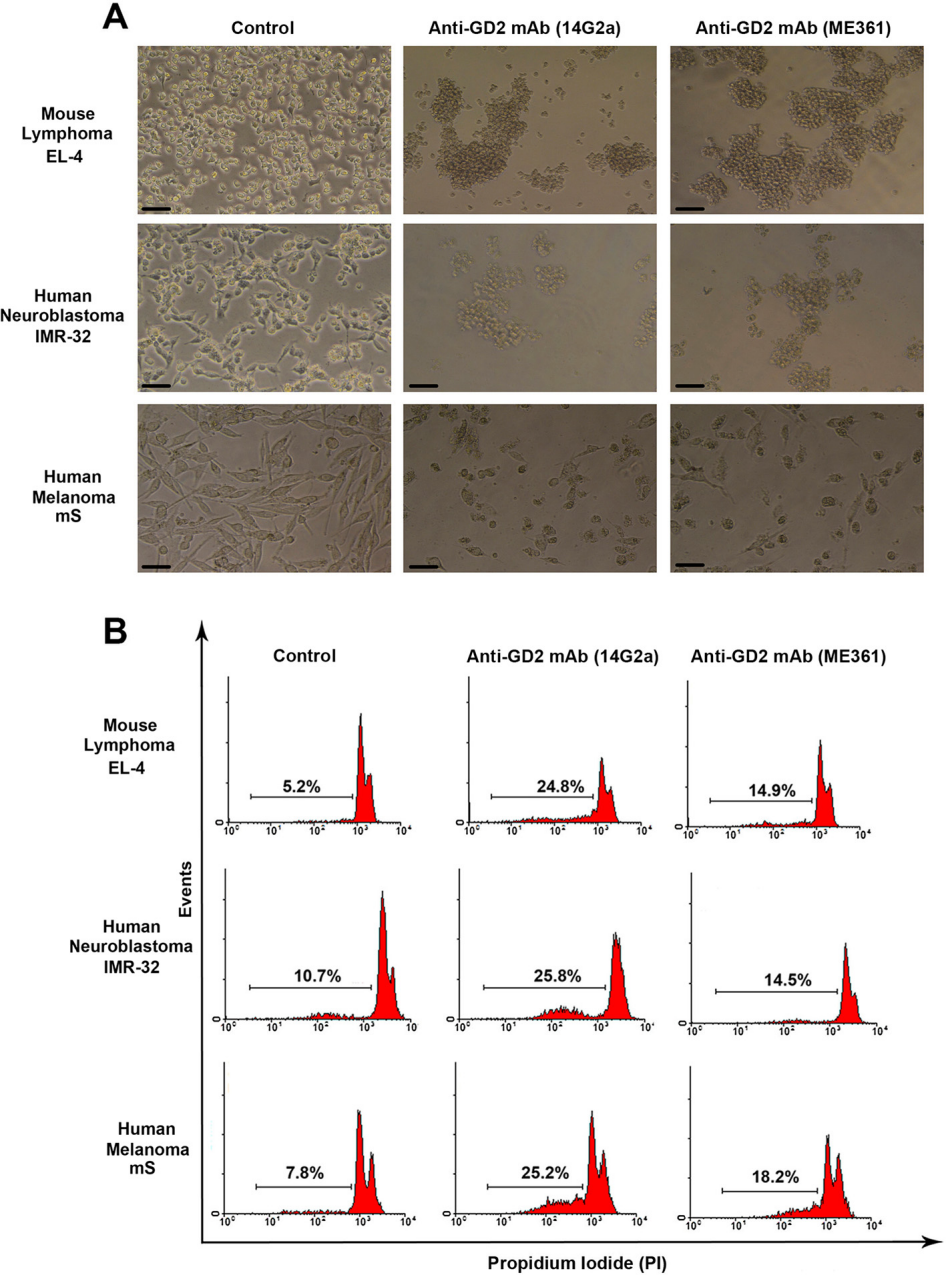
**The cytotoxic effects of two types of anti-GD2 antibodies on GD2-positive tumor cell lines.** Phase-contrast images of GD2-positive tumor cell lines EL-4, IMR-32, and mS after 24 h of incubation with or without anti-GD2 mAbs, 14G2a (5 μg/ml) and ME361 (5 μg/ml) are shown in **(A)**. In **(A)**, bar scale: 50 μm. Analysis of DNA fragmentation (PI assay; see *Methods*) of GD2-positive tumor cells EL-4, IMR-32, mS treated with GD2 mAbs 14G2a (5 μg/ml) and ME361 (5 μg/ml) is shown in **(B)**. In **(B)**, the percentages of the cells with fragmented DNA in hypodiploid peaks are shown for each histogram.

The cytotoxic effects of anti-GD2 mAbs on selected GD2-positive and GD2-negative cell lines were further investigated using two different monoclonal antibodies 14G2a and ME361. We found that after 24 h of incubation of tumor cells with anti-GD2 mAbs at concentration of 5 μg/ml GD2-positive cells underwent significant morphological changes: shrinkage and rounding of the cells, their detachment from plates, and formation of cell aggregates. All of these morphological changes were the most dramatic for GD2-positive EL-4 and mS cell lines (Figure 3A). These anti-GD2 mAbs had no effect on morphology of all examined GD2-negative cell lines (Additional file 2A).

Next, we investigated DNA fragmentation in the population of the cells treated with anti-GD2 mAbs. After incubation with anti-GD2 antibodies, the cells were fixed, permeabilized and stained with DNA-binding dye propidium iodide (PI). The percentage of the cells in hypodiploid peak of three tested GD2-positive tumor cell lines EL-4, mS and IMR-32 was increased after anti-GD2 treatment when compared to untreated cells. After incubation with two different anti-GD2 antibodies 14G2a and ME361 at concentrations of 5 μg/ml the percentage of EL-4 cells with fragmented DNA increased 5.0 ± 0.7 and 3.1 ± 0.9 fold above baseline level, respectively (Figure 2B). When compared to EL-4 cells, an increase in percentage of the cells with fragmented DNA for IMR-32 and mS cell lines was slightly lower, but still statistically significant After incubation with 14G2a and ME361 mAbs, the proportion of IMR-32 cells with fragmented DNA increased 2.5 ± 0.5 and 1.7 ± 0.4 fold, respectively. For mS cells treated with 14G2a and ME361 antibodies, these values were 3.2 ± 0.4 and 2.3 ± 0.5, respectively (Figure 3B). Anti-GD2 mAbs did not affect GD2-negative tumor cell lines (Additional file 2B).

**Figure 4:**
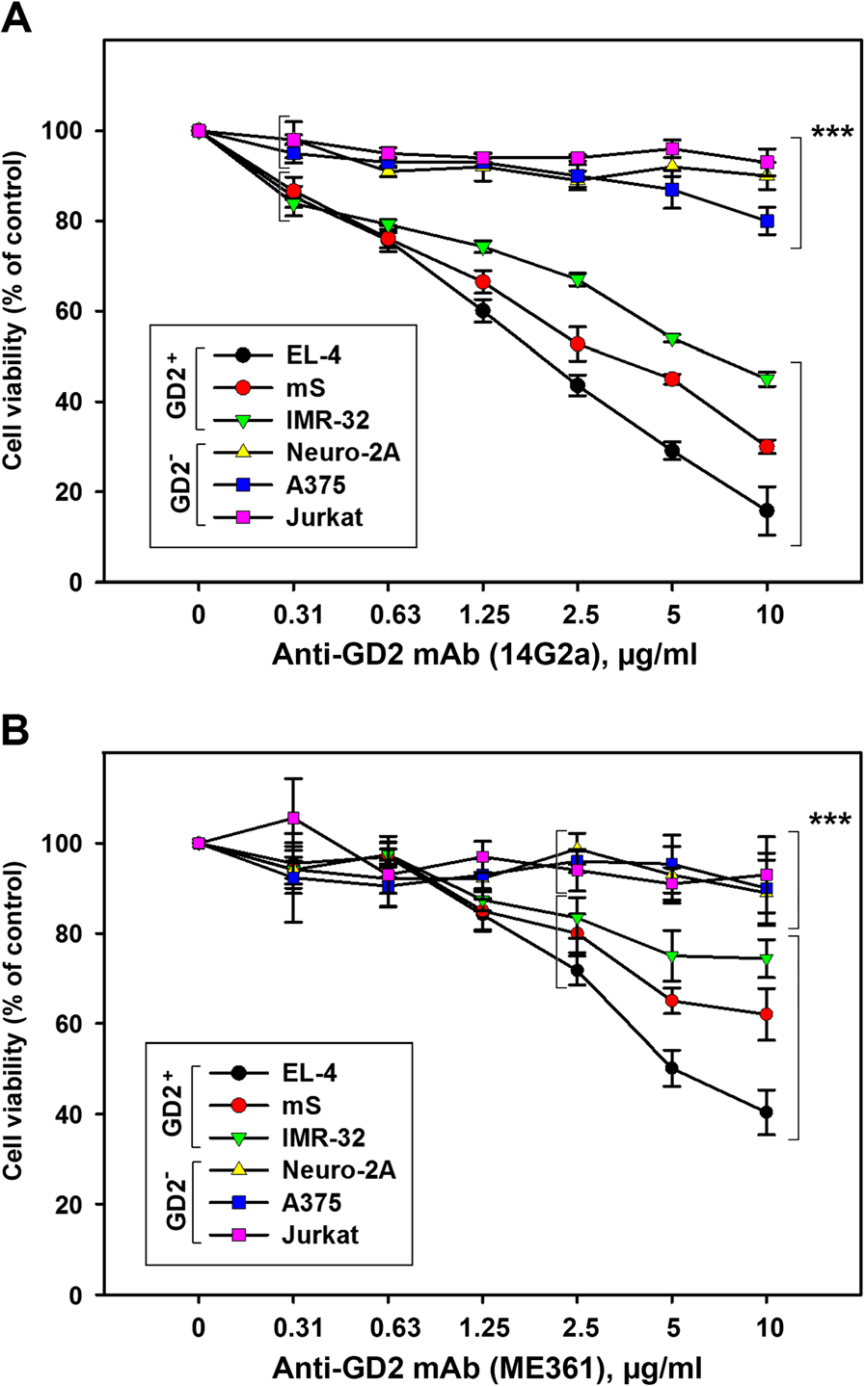
**Comparison of the influence of anti-GD2 antibodies on viability of GD2-positive vs. GD2-negative tumor cell lines.** The viability of GD2-positive (EL-4, IMR-32, mS) and GD2-negative (Neuro-2A, A375, Jurkat) tumor cells was assessed for the cells incubated with various concentration of anti-GD2 mAbs for 72 h using MTT assay as described in *Methods*. Results are shown for two monoclonal anti-GD2 antibodies 14G2a **(A)** and ME361 **(B)**. Mean ± S.E. of three separate experiments is shown, statistical analysis was performed using two-way analysis of variance method for concentrations of 0.31 – 10 μg/ml **(A)**, and for concentrations 2.5 – 10 μg/ml **(B)**. The differences between GD2-positive and GD2-negative groups were statistically significant (***, P < 0.001) as determined by Student-Newman-Keuls post-hoc analysis.

We further investigated the viability of tumor cells incubated with various concentrations of anti-GD2 mAbs using MTT assay. As shown in Figure 4, anti-GD2 antibodies substantially decreased viability of GD2-positive EL-4, mS and IMR-32 cell lines, without a significant influence on GD2-negative cell lines Neuro-2A, A375, and Jurkat. Note that the anti-GD2 antibodies 14G2a were more cytotoxic for GD2-positive cell lines (Figure 4A) when compared to ME361 antibodies (Figure 4B). After 72 h of incubation of the cells with the highest concentration of 14G2a antibodies (10 μg/ml), the strongest effect was observed for EL-4 lymphoma cells, which express the highest level of GD2. While the viability of the EL-4 cells was reduced by more than 80%, the viability of mS and IMR-32 cells decreased by 60-70%. The cytotoxic effect of ME361 antibodies was weaker, but still substantial, and the differences in viability of GD2-positive and GD2-negative cell lines were statistically significant for concentrations of antibodies higher than 2.5 μg/ml (Figure 4B). In case of EL-4 and mS cell lines, the highest concentration of ME361 antibodies of 10 μg/ml decreased the viability of the cells by 60% and 40%, respectively.

These data indicate high level of cytotoxic effects of anti-GD2 mAbs on tumor cells of different origins that express GD2. On the other hand, anti-GD2 mAbs did not influence on GD2-nevative cell lines. At the same time, the cytotoxic activity of two different types of anti-GD2 monoclonal antibodies was variable with the strongest effect displayed by 14G2a antibodies. GD2-positive cell lines varied in their susceptibility to cytotoxic effect of anti-GD2 antibodies with the effect on EL-4 cells being the strongest.

### Anti-GD2 antibodies induce rapid cell death that combined features of apoptosis and necrosis

**Figure 5:**
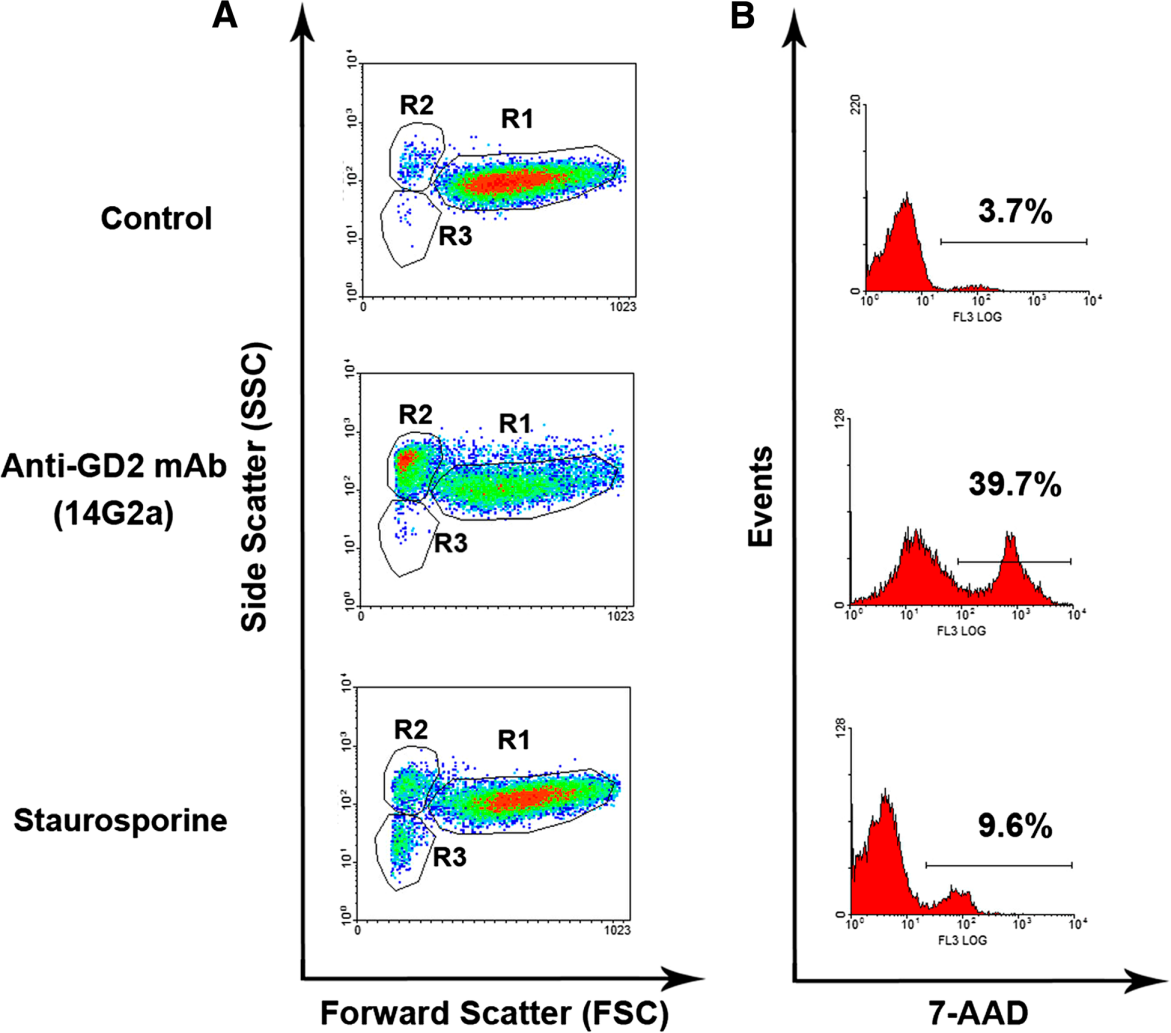
**Analysis of apoptotic volume decrease and the loss of plasma membrane integrity for EL-4 lymphoma cells treated with anti-GD2 antibodies.** Apoptotic volume decrease (AVD) **(A)** and cell membrane permeability **(B)** were analyzed for the control (untreated) EL-4 cells or after 2 h of incubation with anti-GD2 mAbs 14G2a (5 μg/ml), or Staurosporine (500 nM) that was used as positive control for induction of apoptosis (see *Methods*). In **(A)**, R1 – region of viable cells, R2 – region of cells with AVD, and R3 – region of cell debris. In **(B)**, percentages of 7-AAD positive cells are shown for each histogram.

**Figure 6:**
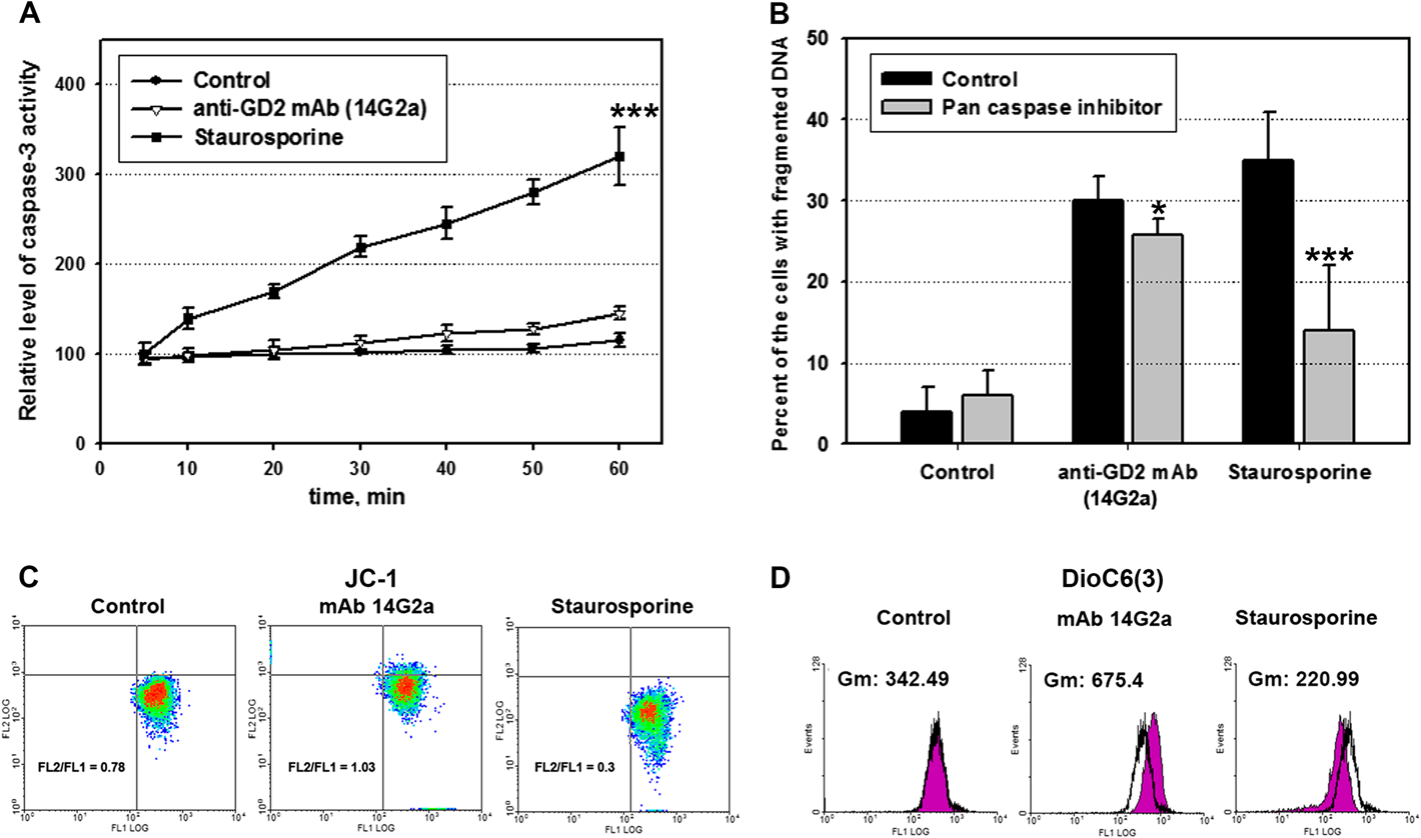
**Analysis of caspase-3 activation and mitochondria involvement during the cell death induced by anti-GD2 antibodies.** Enzymatic activity of caspase-3 in control (untreated) EL-4 cells, or treated with anti-GD2 mAbs 14G2a (5 μg/ml), or staurosporine (50 nM) for 24 h is shown in **(A)**. Mean ± S.E. of three separate experiments is shown. The statistical analysis was performed using two way analysis of variance method. There was a statistically significant differences between groups (P ≤ 0.001), *** P < 0.001 as determined by multiple comparisons of experimental versus control groups using Dunnett's post-hoc analysis. Effect of Pan-caspase inhibitor Z-VAD-FMK (10 μM) on cell death induced by anti-GD2 mAbs 14G2a (5 μg/ml) and Staurosporine (50 nM) after 24 h of incubation with EL-4 cells is shown in **(B)**. Statistical analysis was performed using Mann–Whitney rank sum test, the differences between control and pan-caspase inhibitor groups were statistically significant (*, P < 0.05; ***, P < 0.001). Effect of anti-GD2 mAbs 14G2a (5 μg/ml) and staurosporine (50 nM) on ΔΨm of AVD-positive and 7AAD-negative populations of EL-4 cells. Representative density plots of flow cytometry analysis of mitochonodrial potential (MPT) measured by using JC-1 probe (2 μg/ml) in intact versus the cells incubated with anti-GD2 mAbs 14G2a (5 μg/ml) or staurosporine (500 nM) for 2 h is shown. **(C)**. Representative density plots of MPT measured by using DioC_6_(3) probe (20 nM) in intact and cells incubated with anti-GD2 mAbs 14G2a (5 μg/ml) or staurosporine (500 nM) for 2 h is shown **(D)**.

We have chosen EL-4 cells and monoclonal antibody 14G2a as an optimal model to study mechanisms of cell death induced by anti-GD2 mAb. We found that after incubation of EL-4 cells with anti-GD2 mAb 14G2a there was a significant increase in the proportion of the cells with apoptotic volume decrease (AVD) (Figure 5A; *14G2a*; gate R2) and the cells with permeable cell membrane (Figure 5B; *14G2a*). After 2 h of cell exposure to anti-GD2 antibodies, 35 ± 6% of cells exhibited AVD (Figure 5A; *14G2a*; gate R2), and 40 ± 4% cells exhibited permeability of cell membrane as determined by 7-AAD incorporation (Figure 5B; *14G2a*). At the same time, only 4-8% of the cells with AVD were found in the control untreated cells and only 3.5-7% of untreated cells were 7-AAD-positive (Figure 5, *control*). We used staurosporine as positive control for cell death induction. The effect of staurosporine was less dramatic than the effect of antibodies: 7-10% of AVD cells (Figure 5A, *staurosporine*; gate R2), and 8-11% of 7-AAD positive cells (Figure 4B, *staurosporine*). Next we investigated activation of caspase-3 in EL-4 cells treated for 24 h with anti-GD2 antibody 14G2a using fluorescently labeled substrate for caspase-3 Z-DEVD-AFC. We found that anti-GD2 antibodies did not cause substantial activation of caspase-3: the level of activity of this effector caspase was 3–4 folds lower for anti-GD2-treated cells when compared to the EL-4 cells treated with staurosporine (Figure 6A). Pan-caspase inhibitor Z-VAD-FMK did not have any significant effect on cell viability induced by anti-GD2 antibodies, but it did decrease (2.7-fold) the percentage of apoptotic cells treated with staurosporine (Figure 6B).

We further analyzed the mitochondria involvement in the cell death induced by anti-GD2 mAb using two specific sensitive fluorescent probes JC-1 and DiOC_6_(3). Flow cytometry analysis of mitochondrial membrane potential (MMP) of AVD- and 7-AAD-negative EL-4 cells was performed and the results are shown in Figure 6C, D. Using JC-1 and DiOC_6_(3) probes, we found that treatment of cells with anti-GD2 mAb 14G2a for 2 h resulted in a significant increase in ΔΨm as determined by increased ratio of FL2/FL1 fluorescence for JC-1 (Figure 6C) and increase in MFI of green fluorescence (FL1 channel) in 14G2a-treated cells for DiOC_6_(3) (Figure 6D) when compared with ΔΨm of intact cells. At the same time, staurosporine induced depolarization of MMP in the AVD- and 7-AAD-negative cell populations (Figure 6C and D). We found that there was a significant decrease in MMP in AVD- and 7-AAD-positive populations when compared with AVD- and 7-AAD-negative populations of the cells treated with anti-GD2 mAb, staurosporine, or untreated control cells (data not shown). Thus, we suggested that the first event of anti-GD2 mAb-induced cell death was a hyperpolarization of mitochondrial membrane potential, and then AVD, cell membrane permeability and decrease in MMP were occurred.

These results indicated that anti-GD2 mAb induced non-classical mitochondria-dependent cell death with the features of both apoptosis and necrosis and that caspases did not play a pivotal role in this process.

### Cross-reactivity of anti-GD2 mAbs with cell adhesion molecule ALCAM and other gangliosides

**Figure 7:**
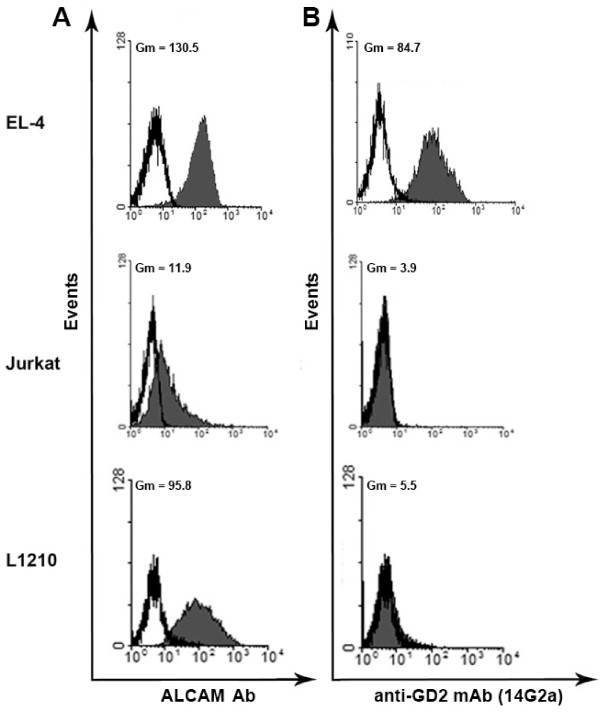
**Cross-reactivity of anti-GD2 antibodies with ALCAM adhesion molecule.** Flow cytometry analysis of EL-4, Jurkat and L1210 cells stained with anti-ALCAM (С20) antibodies is shown in **(A)**, and staining with anti-GD2 14G2a antibodies is shown in **(B)** . In **(A, B)** filled histograms show staining with anti-GD2 mAbs, empty histograms – staining with secondary control antibodies.

There is an evidence that 14G2a antibodies could cross-react with highly glycosylated ALCAM (CD166) adhesion molecule [20], which is expressed in different tissues, mainly on cells of the immune system, and this molecule does not exhibit tumor association. In our experiments, Western blot analysis showed that anti-GD2 antibodies 14G2a could bind to certain protein with a molecular weight of 105–115 kDa from lysate of EL-4 cells. At the same time anti-GD2 antibodies ME361 did not react with any protein from the same EL-4 cell lysate (not shown). Although 14G2a antibodies reacted with the protein that has a molecular weight similar to ALCAM (100–105 kDa), these results do not provide ultimate evidence that 14G2 antibodies react with ALCAM, but not with other proteins of the similar weight. Moreover, even if such interaction of 14G2 antibodies with ALCAM is confirmed, it does not necessarily indicate that 14G2a mAb specifically interacts with extracellular part of ALCAM molecule. To assess the possibility of interaction of 14G2a with extracellular part of ALCAM molecule, we have selected several cell lines that expressed ALCAM and, at the same time, were negative for GD2 (Figure 7).

Using specific antibodies that recognize extracellular C-terminus of the ALCAM molecule we demonstrated that GD2-positive cell line (EL-4) and two GD2-negative cell lines (Jurkat and L1210) expressed ALCAM on their surface (Figure 7A). At the same time, staining of Jurkat and L1210 cells with anti-GD2 antibodies 14G2a demonstrated that these antibodies did not bind to these ALCAM positive cells (Figure 7B). We concluded from these experiments that 14G2a antibodies did not bind the extracellular region of ALCAM on the surface of ALCAM-positive cell lines.

**Figure 8:**
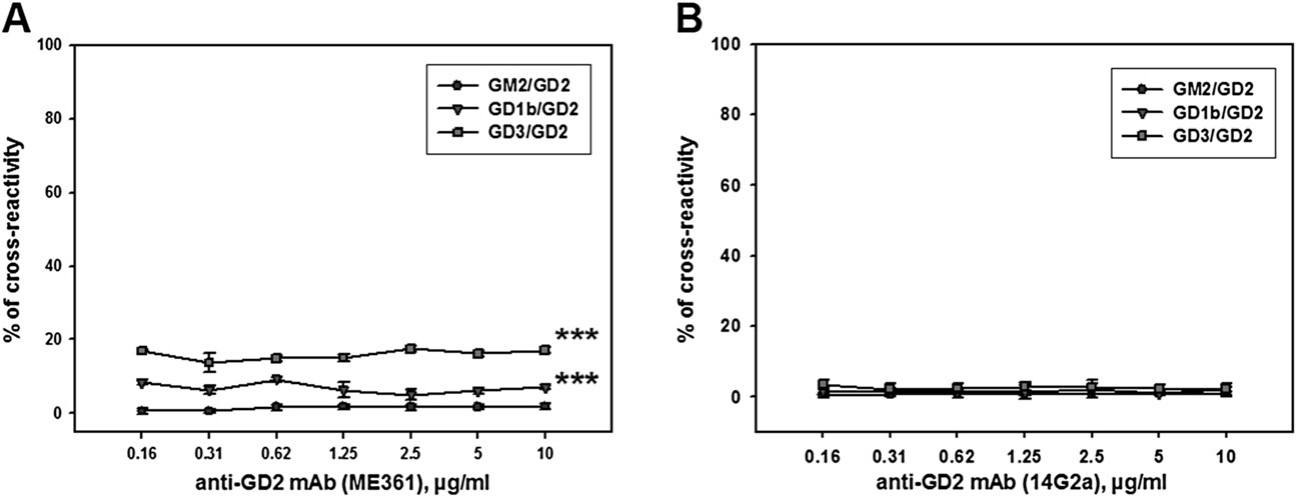
**Cross-reactivity of anti-GD2 antibodies with gangliosides GM1, GM2, GD1b and GD3. (A, B)** Interaction of anti-GD2 antibodies with gangliosides GD2 vs. GM1, GM2, GD1b and GD3 was assessed by ELISA as described in *Methods*. Plates were coated with gangliosides GD2, GM1, GM2, GD1b, and GD3 (0.25 μg/well) and incubated with two types of anti-GD2 mAbs (0.156 - 10 μg/ml) ME361 **(A)** and 14G2a **(B)**. In **(A),** the level of cross-reactivity is presented as the ratio for GM2, GD1b and GD3 binding to that of GD2. Mean ± S.E. of three separate experiments are shown, statistical analysis was performed using two-way analysis of variance method. There was a statistically significant difference between groups (P ≤ 0.001), ***P <0.001 as determined by multiple comparisons versus zero cross-reactivity by Dunnett's post-hoc analysis **(A)**. In **(B),** multiple comparisons were not performed, since the values of the cross-reactivity were less than 2%.

Due to similar structure of various types of gangliosides, it was also important to evaluate the ability of anti-GD2 mAbs 14G2a and ME361 to cross-react with other gangliosides. We evaluated binding properties of both monoclonal antibodies 14G2a and ME361 to immobilized gangliosides by ELISA. BODIPY-FL-C5-labeled gangliosides were used to check amounts of gangliosides adsorbed to the plate to ensure equal amount of gangliosides (0.3 ng/well) in each well for further ELISA analysis (Additional file 3). This assay allowed us to conduct a quantitative comparison of binding patterns of anti-GD2 mAbs 14G2a and ME361 to various gangliosides. Our analysis of cross-reactivity of anti-GD2 mAbs is presented in Figure 8A, B. The ME361 antibody displayed a weak cross-reactivity with ganglioside GD3 (14-17% of their binding to GD2) and GD1b (5-9% of their binding to GD2) (Figure 8A), while 14G2a antibodies showed no significant cross-reactivity with the gangliosides GM2, GD1b and GD3 (Figure 8B). Consequently, the cytotoxic effects of ME361 antibodies could be also mediated by interaction with not only GD2, but also with gangliosides GD1b and GD3. However selected for these experiments EL-4 cells did not have any detectable levels of gangliosides GD3 or GD1b in the total ganglioside content (Figure 2A). Flow cytometry analysis of EL-4 cells stained with anti-GD3 mAb MB3.6 further confirmed that GD3 is not expressed on the cell surface of these cells (not shown). Since gangliosides GD3 and GD1b are not expressed on EL-4 cells, ME361 mAb could only bind to ganglioside GD2 on the surface of these cells to induce cell death. Thus, our results indicate that two of our monoclonal anti-GD2 antibodies, 14G2a and ME361, mediated cytotoxic effect in EL-4 cells by interacting specifically with GD2 but not with glycoproteins or other gangliosides.

### Correlation of the GD2 expression level with susceptibility of the cells to anti-GD2 mediated cell death

**Figure 9:**
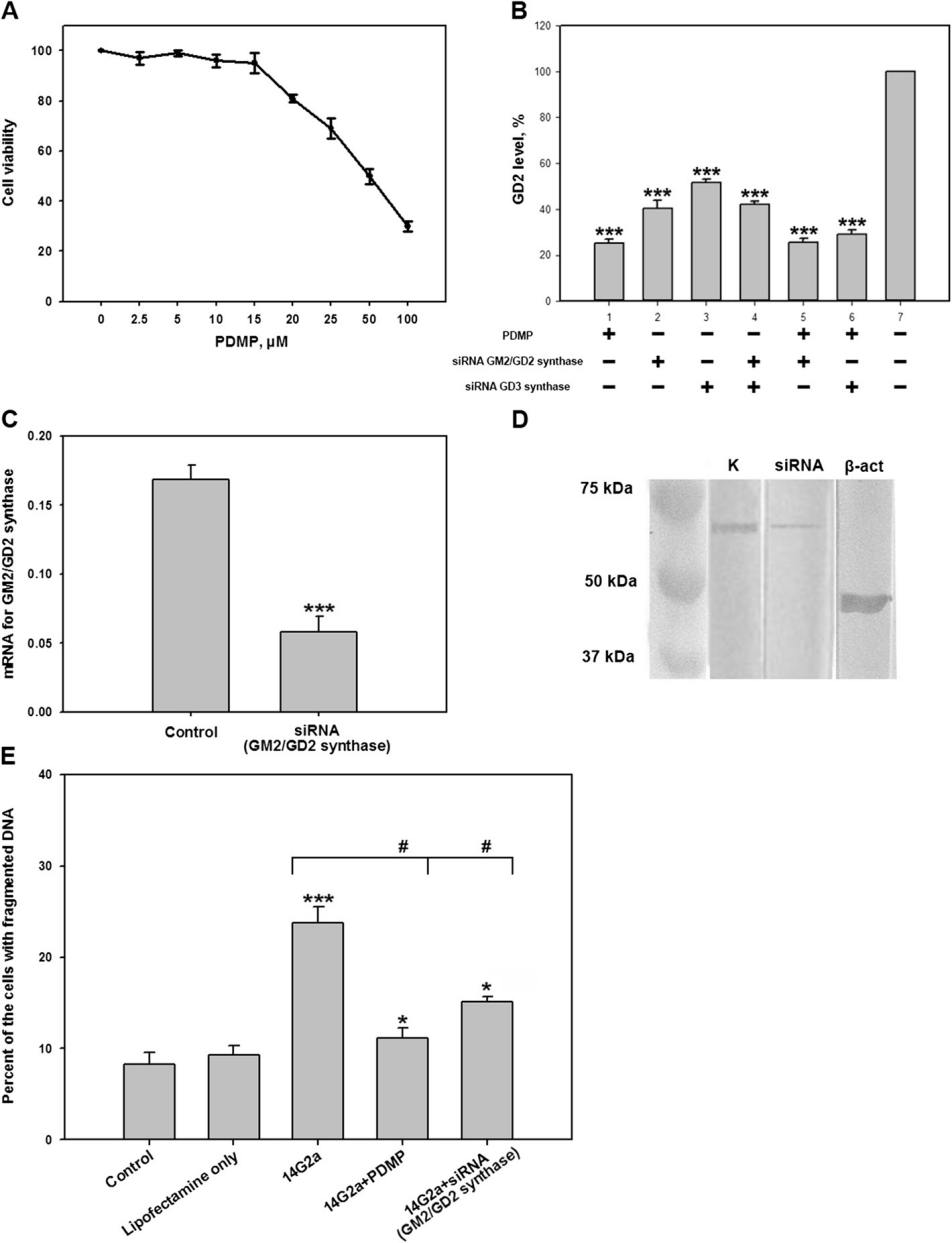
**Analysis of susceptibility of EL-4 cells with down-modulated GD2 to cell death induced by anti-GD2 antibodies.** Cell viability (MTT assay, see *Methods*) of EL-4 cells treated with various concentrations of PDMP (2.5-100 μM) is shown in **(A)**. Flow cytometry analysis of GD2 expression in the control cells vs. the cells with inhibited GD2 biosynthesis (with PDMP or siRNA GM2/GD2 synthase) is shown as the ratio of MFI of cells with reduced GD2 expression to MFI of control cells **(B)**. The expression of GM2/GD2 synthase on mRNA level is shown in **(C)**. The expression of GM2/GD2 synthase on a protein level in EL-4 cells transfected with siRNA that target GM2/GD2 synthase is shown in **(D)**. Cytotoxic effects of anti-GD2 mAb 14G2a (5 μg/ml) on control EL-4 cells vs. cells with inhibited GD2 expression is shown in **(E)**. In **(C)**, RNA from control cells and cells transfected with GM2/GD2 synthase siRNA was isolated, and the expression of mRNA for GM2/GD2 synthase was determined by real time RT-PCR as described in *Methods*. In (D), expression of GM2/GD2 synthase was analyzed by Western blot as described in *Methods*. In **(E)**, cytotoxicity was analyzed by PI assay as described in *Methods*.

One of the experimental approaches to reduce of GD2 expression on the cell surface is the usage of the common ganglioside biosynthesis inhibitor PDMP. In the first series of experiments, we determined the optimal concentration of PDMP in order to effectively inhibit ganglioside expression in EL-4 cells without affecting cell viability. The viability and cell death of EL-4 cells treated with PDMP was assessed using MTT- and PI-assays, respectively. We found that 15 μM was the optimal concentration for PDMP that did not affect viability of the EL-4 cells (Figure 9A) and did not induce cell death (not shown). At the optimal concentration of PDMP (15 μM), the level of GD2 expression was reduced by 75% when compared to untreated cells (Figure 9B; *PDMP*).

Another approach to inhibit the biosynthesis of ganglioside GD2 was a transfection of EL-4 cells with siRNA that target GM2/GD2 and GD3 synthases. Transfection of the cells with siRNA for GM2/GD2 synthase resulted in substantial decrease in expression of this enzyme on the mRNA (Figure 9C) and protein (Figure 9D) levels. As shown in Figure 8B, the transfection of the cells with siRNA for GM2/GD2 synthase resulted in ~60% decrease in GD2 expression on the surface of EL-4 cells. Transfection of the cells with siRNA for GD3 synthase resulted to 50-55% decrease in GD2 surface expression level (Figure 9B). Cotransfection of EL-4 cells with two siRNAs for both GM2/GD2 and GD3 synthases did not lead to further decrease in GD2 level. In addition, combination of treatment of EL-4 cells with PDMP and transfection with siRNA for GM2/GD2, or GD3 synthase, did not result in robust decrease in GD2 level when compared with PDMP treatment alone (Figure 9B). This complex treatment with siRNA and PDMP induced significant lost of viability of EL-4 cells (not shown). Therefore, for comparative analysis of cytotoxic effects of anti-GD2 mAb on cells with normal and inhibited GD2 expression, we used cells treated with PDMP or transfection with siRNA for GM2/GD2 synthase, because these treatments were effective for decrease in GD2 level without affecting cell viability.

Using the PI assay we have demonstrated that EL-4 cells with inhibited biosynthesis of ganglioside GD2 were significantly less susceptible to cell death induced by anti-GD2 mAbs. Cells treated with PDMP became irresponsive to anti-GD2 mAbs, while knockdown of GM2/GD2 synthase decreased the percentage of cells with fragmented DNA by ~60% (Figure 9E). These results indicate that susceptibility of the cells to cell death induced by anti-GD2 antibodies directly correlated with the level of GD2 expression at the cell surface.

## Discussion

In this study we demonstrated for the first time a functional role of anti-GD2 antibodies as direct inducers of cells death due to specific binding of these antibodies to GD2. The role of anti-GD2 antibodies has never been examined systematically because of certain technical limitations. In previous publications a limited number of tumor cell lines were used and anti-GD2 antibodies of various origin and broad specificity were utilized that could react not only with GD2 but also with number of other glycoproteins and glycolipids. Therefore it was not known whether GD2 is a single functional molecule that induces cytotoxic signal or other molecules are involved in this process. This fact is especially important, because several studies have shown the anti-GD2 mAbs exhibited cross-reactivity with other structurally similar gangliosides [21], as well as with several cell adhesion molecules [20,22].

In a number of studies it was shown that antibodies against various tumor-associated gangliosides have tendency to induce or enhance the tumor cells death [9,11,23-25]. It is worth noting that Fab-fragments of these antibodies retain the functional activity of whole molecules to induce the cell death, and thus the cross-linking of gangliosides on the cell surface by the whole antibody molecules (or interaction with immune cells or complement via Fc-fragments) is not essential for the induction of cell death [12,26]. This fact suggests that gangliosides may have the ability to accept and transduce the signals of the death inside the cell. However, glycosphingolipids, particularly gangliosides, do not belong to classical death receptors (e.g. CD95) since they are lipid, not protein molecules, and lack the classic transmembrane domain capable of signal transduction. However, gangliosides could serve as the target molecules for a number of ligands due to their specific localization in the outer monolayer of plasma membrane and due to the presence of branched sialylated carbohydrate chains exposed at the extracellular space. For example, the ganglioside GM1 specifically binds cholera toxin B-subunit [27], and gangliosides GD1a, GD1b, GT1b bind tetanus and botulinum toxins [28,29]. But in these cases, gangliosides are considered to be just passive binding receptors that do not transduce signal inside the cell. On the other hand, gangliosides were found to be involved in cell death processes such as apoptosis. It was demonstrated that intracellular level of the ganglioside GD3 is increased during progression of apoptotic signal induced through CD95, and inhibition of GD3-synthase leads to reduction of the apoptosis [30]. In several instances it was reported that gangliosides may act as not only modulators but also inducers of cell death. For example, exogenous monosialic gangliosides induced apoptosis in CD8 T cells, which was considered to be one of the major mechanisms of immune suppression mediated by the tumor-associated gangliosides [31].

As the functions of gangliosides in regulation of cell death and the importance of GD2 as a target molecule for antibody-based anti-cancer therapy are not well defined, we believe that it is important to evaluate the role of GD2 in the reception and transduction of the cytotoxic signal. For this purpose, we used two different monoclonal antibodies against ganglioside GD2. Using anti-GD2 antibodies ME361 and 14G2a, we showed that these antibodies specific for ganglioside GD2 could induce strong cytotoxic effects on tumor cells of different origins. We established a number of common features of effects anti-GD2 mAbs on various tumor cell types. First, cytotoxic effects of the antibodies were observed only for GD2-positive cells. Second, the strongest cytotoxic effects were observed in the case of EL-4 lymphoma, which is characterized by the highest level of GD2 expression in comparison with other cell lines. Third, both anti-GD2 mAbs 14G2a and ME361 substantially decreased viability of EL-4, mS and IMR-32 cell lines in a dose-dependent manner. Thus, these results confirm the involvement of ganglioside GD2 in the reception and transduction of cytotoxic signal.

As we mentioned above, the studying of functional role of gangliosides such as GD2 is challenging due to cross-reactivity of anti-GD2 mAbs with a number of glycosylated proteins and other glycosphingolipids with a similar structure of the carbohydrate chains [20,32]. For example, gangliosides GD3 and GD1b have rather similar structures. In addition, a sialylated adhesion molecule NCAM has the same type of sialic acid linkages as ganglioside GD2. In the study by Patel et al. [22] it was suggested that anti-GD2 monoclonal antibody 3 F8 recognizes not only GD2 but also NCAM. Furthermore, the same binding sites for anti-GD2 mAbs may be present on other glycoproteins that do not have the structural similarity with GD2. It was shown that the anti-GD2 mAb 14G2a could interact with another adhesion molecule ALCAM, which is structurally similar to NCAM [20]. On the other hand, reported interactions of anti-GD2 antibodies with ALCAM and/or NCAM is debatable since it was not confirmed by other researchers [33,34]. In view of these conflicting results, we conducted series of experiments to examine possible cross-reactivity anti-GD2 mAbs with other molecules structurally related to GD2 and also expressed in tumor cells. In these experiments we have shown that ME361 did not bind to any proteins from whole cell lysate of EL-4 cells, while 14G2a could bind to the protein of a molecular weight of 105–110 kDa, which is consistent with results published by Kozber et al. [20]. However, the results of our flow cytometry analysis of reactivity of 14G2a mAbs with GD2-negative/ALCAM-positive cell lines have shown that although cross-reactivity of 14G2a with ALCAM was obvious as determined by Western blot analysis. However, we found that the binding site of the protein exposed to the antibodies in Western blot is not located on the extracellular portion of ALCAM molecule as determined by FACS. Therefore, ALCAM does not play a significant role for death signal transduction triggered by anti-GD2 mAb 14G2a. In our study of cross-reactivity of anti-GD2 mAbs with other gangliosides, we found that ME361, which exhibit high affinity to ganglioside GD2, could bind gangliosides GD1b and GD3. For 14G2a antibodies, the cross-reactivity with other gangliosides was not detected. We found that EL-4 lymphoma express only gangliosides GD2 and GM2. Since the gangliosides GD3 and GD1b are absent in the cell membrane of EL-4 cells, the anti-GD2 mAb ME361 could only interact with ganglioside GD2, and cross-reactivity of these antibodies with other gangliosides did not contribute to observed cytotoxic effect of these antibodies. Thus, our results point out the predominant role of GD2 in the reception of cytotoxic signals induced by two types of anti-GD2 antibodies.

To further prove the exclusive role of ganglioside GD2 in a reception of cytotoxic signal, we reduced the expression level of GD2 on the surface of EL-4 tumor cell line, and these cells with decreased expression of GD2 were used to study cytotoxic effects of anti-GD2 mAbs. Comparison of the efficiency of anti-GD2-mAb-induced cytotoxic effects in intact cells versus cells with reduced expression of GD2 allowed us to directly assess the contribution of this ganglioside in induction of cell death. Inhibition of the enzymes responsible for the ganglioside synthesis let us to obtain the cells with significantly reduced expression of GD2. The cytotoxic effect caused by anti-GD2 mAbs was much higher for intact cells than for cells with inhibited expression of GD2. Thus, we demonstrated that ganglioside GD2 itself could serve as a receptor of cell death in GD2-positive tumor cells.

However, several important questions remain unclear: 1) how does the GD2 transmit a cell death signal inside the cell; 2) what causes the variability in efficiency of the cytotoxic effect induced by the different anti-GD2 mAbs; 3) whether the property of GD2 molecule being a receptor of death signal is a general feature of other tumor-associated gangliosides?

The published reports regarding the role of gangliosides in regulation of cell death are rather conflicting and contradictory. The researchers have incoherent points of view about the mechanisms of the cytotoxic action of antibodies to the tumor-associated gangliosides. Thus, a number of researchers have observed certain aspects of apoptotic cell death (e.g. activation of caspases and other typical characteristics of classic apoptosis) in the cells exposed to the GD2- and NeuAcGD2-specific antibodies [10,25,35]. On the other hand, it was shown that antibodies to tumor-associated ganglioside NeuGcGM3 induced cell death by mechanism of necrosis with formation of the membrane pores. The researchers showed that this process is caspase-independent [24,36]. Such results could be explained by both the different origin of tumor cell lines used in these studies, and by targeting different tumor-associated gangliosides. Also it was reported that caspases did not play a key role and did not determine the mechanism of cell death triggered by anti-GD2 mAbs [37]. According to this study, cell death signaling pathways triggered by anti-GD2 mAbs are complex and could be attributed to non-classical mechanisms of cell death, with features of apoptosis (e.g. AVD) and necrosis (e.g. plasma membrane permeability), and with involvement of mitochondria-dependent pathways.

We assume that one of the mechanisms of the observed process induced by anti-GD2 mAb is the translocation of ganglioside GD2 from cell membrane into intracellular compartments that leads to change in intracellular traffic resulting in mitochondria damage. As shown in our study, the anti-GD2 mAb induced rapid hyperpolarization of mitochondrial membrane potential. We also detected rapid internalization of the complexes of anti-GD2 mAbs with ganglioside GD2 across the cell membrane inside the cell. For 14G2a antibodies this process was more effective compared to ME361 mAbs (unpublished observation). On the other hand, it is known that induction of cell death through classic death receptors such as Fas/CD95 or TNFR results in changing of the intracellular traffic and/or biosynthesis of GD3, a ganglioside structurally similar to GD2, leading to translocation of GD3 into the mitochondria and to direct induction of cell death in a mitochondria-dependent manner [30,38]. We suggest that binding of anti-GD2 mAbs with GD2 on a cell surface could lead to translocation of this ganglioside into mitochondria and induction of cell death in a manner similar to GD3.

Thus, our study suggests new mechanisms of direct cytotoxic action of ganglioside-specific antibodies, which are different from classical apoptosis and require further investigation.

## Conclusions

We provided evidence for the new functional activity of GD2 ganglioside as a receptor for cell death signal. Since GD2 is a promising target of anti-cancer therapy, the observed effector properties of GD2 as a receptor and transducer of death signal could be used for the development of new types of anti-cancer drugs.

## Competing interests

The authors declare that they have no competing interests.

## Authors' contributions

RVK conceived the idea; IID, PAV, RVK, IMM and DYR performed the experiments; RVK, IVK and IMM designed the experiments and analyzed the data. RVK, IVK and EDP performed further data analysis and interpretation and wrote the manuscript. All authors read and approved the manuscript.

## Pre-publication history

The pre-publication history for this paper can be accessed here:

http://www.biomedcentral.com/1471-2407/14/295/prepub

## Supplementary Material

Additional file 1 **Expression of GD2 on the cell surface of Jurkat, Neuro-2a, and A375 tumor cell lines.** Flow cytometry analysis of the cells stained with anti-GD2 antibodies conjugated with AlexaFluor488 (14G2a antibodies; 5 μg/ml; see *Methods*) is shown in **(A)**. Filled histograms (red color) show staining with anti-GD2 mAbs, empty histograms – staining with an isotype control. Confocal imaging of Jurkat, Neuro-2a, and A375 cells stained with anti-GD2 conjugated with AlexaFluor488 (14G2a antibodies; 5 μg/ml; see *Methods*) is shown in **(B)**. The nuclei were counterstained with Hoechst 33342 (shown in blue). In **(B)**, bar scale: 50 μm. Click here for file

Additional file 2 **The cytotoxic effects of anti-GD2 antibodies on GD2-negative tumor cell lines.** Phase-contrast images of GD2-negative tumor cell lines Jurkat, Neuro-2a, and A375 after 24 h of incubation with or without anti-GD2 mAbs, 14G2a (5 μg/ml) and ME361 (5 μg/ml) are shown in (**A)**. Analysis of DNA fragmentation (PI assay; see *Methods*) of GD2-negative tumor cell lines Jurkat, Neuro-2a, and A375 treated with GD2 mAbs 14G2a (5 μg/ml) and ME361 (5 μg/ml) is shown in **(B)**. In **(A)**, bar scale: 50 μm. In **(B)**, percentages of the cells with fragmented DNA in hypodiploid peaks are shown for each histogram. Click here for file

Additional file 3 **Quantitative analysis of the gangliosides adsorbed on the ELISA plates. (A)** The RFU (relative fluorescence units) level of fluorescent-labeled gangliosides BODIPY-FL-C5-GM1 and BODIPY-FL-C5-GD3 bound to the well before TMB reaction development in ELISA experiments is shown. Mean ± S.E. of nine separate experiments is shown. The RFU level was measured at 490 nm. The amount of BODIPY-FL-C5-GM1 bound to the well = 0.29 ± 0.04 ng, BODIPY-FL-C5-GD3 = 0.34 ± 0.05 ng which was measured using calibration curve. Statistical analysis was performed using Student’s t-test, there was not a statistically significant difference between BODIPY-FL-C5-GD3 and BODIPY-FL-C5-GM1groups (P = 0.765). **(B)** Calibration curve of fluorescent-labeled gangliosides BODIPY-FL-C5-GM1 and BODIPY-FL-C5-GD3 is shown, Linear regression: RFU BODIPY-FL-C5-GD3 = 20.726 + (271.329 × Amount of ganglioside per well), RFU BODIPY-FL-C5-GM1 = 36.396 + (248.714 × Amount of ganglioside per well, RFU - relative fluorescence units). Titration points are shown as Mean ± S.E of nine experiments. Statistical analysis was performed using Mann–Whitney rank sum test, there was no statistically significant difference between BODIPY-FL-C5-GD3 and BODIPY-FL-C5-GM1 (P = 0.911; not significant). Click here for file
