# Supplementary material for: Ganglioside GD2 in reception and transduction of cell death signal in tumor cells
Source: BMC Cancer. 2014 Apr 28;14:295. doi: 10.1186/1471-2407-14-295 (PMC4021548; doi:10.1186/1471-2407-14-295)
Supplement: Additional file 1 — Expression of GD2 on the cell surface of Jurkat, Neuro-2a, and A375 tumor cell lines. Flow cytometry analysis of the cells stained with anti-GD2 antibodies conjugated with AlexaFluor488 (14G2a antibodies; 5 μg/ml; see Methods) is shown in (A). Filled histograms (red color) show staining with anti-GD2 mAbs, empty histograms – staining with an isotype control. Confocal imaging of Jurkat, Neuro-2a, and A375 cells stained with anti-GD2 conjugated with AlexaFluor488 (14G2a antibodies; 5 μg/ml; see Methods) is shown in (B). The nuclei were counterstained with Hoechst 33342 (shown in blue). In (B), bar scale: 50 μm. [file 1471-2407-14-295-S1.pdf]

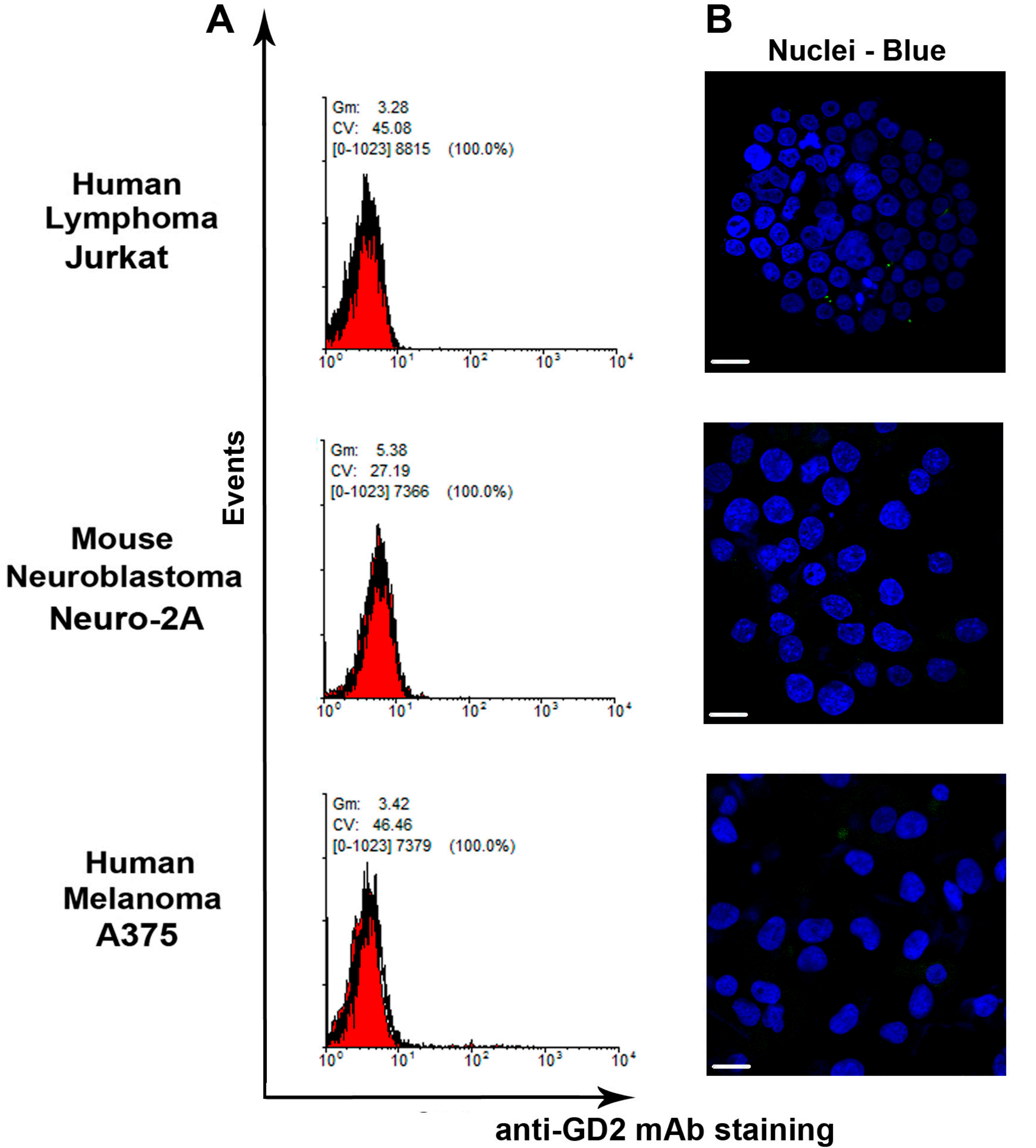
A
B
Nuclei - Blue
Human
Lymphoma
Jurkat
Gm: 3.28
CV: 45.08
[0-1023] 8815 (100.0%)
Mouse
Neuroblastoma
Neuro-2A
Gm: 5.38
CV: 27.19
[0-1023] 7366 (100.0%)
Human
Melanoma
A375
Gm: 3.42
CV: 46.46
[0-1023] 7379 (100.0%)
Events
10^0
10^1
10^2
10^3
10^4
anti-GD2 mAb staining
