# Supplementary figures and images for: Ganglioside GD2 in reception and transduction of cell death signal in tumor cells
Source: BMC Cancer. 2014 Apr 28;14:295. doi: 10.1186/1471-2407-14-295 (PMC4021548; doi:10.1186/1471-2407-14-295)

**A**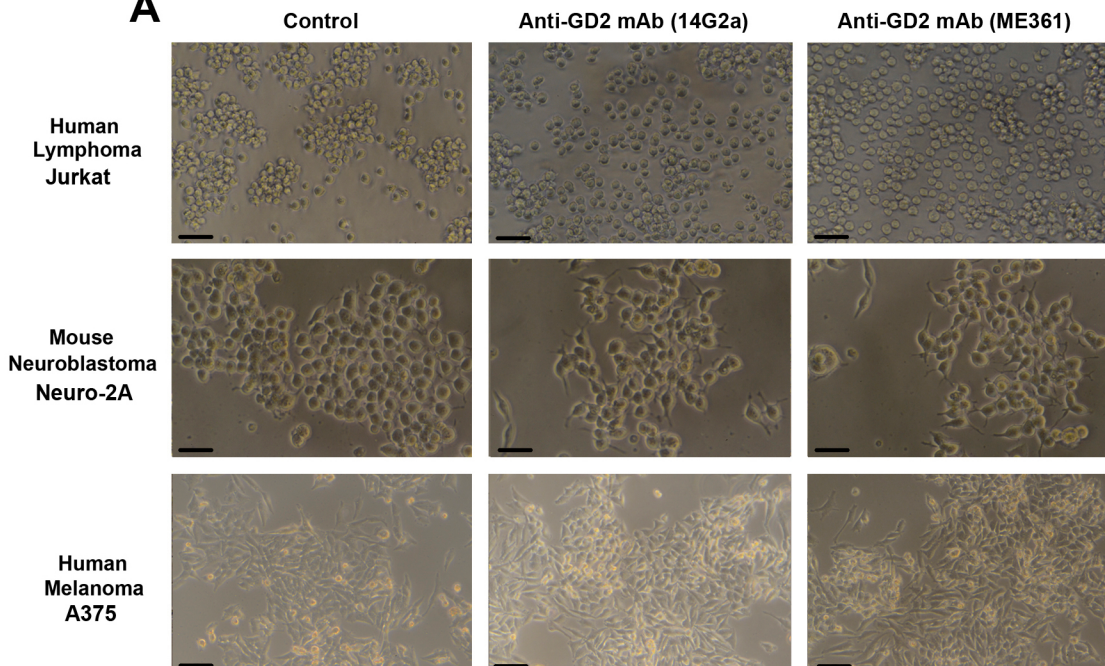**B**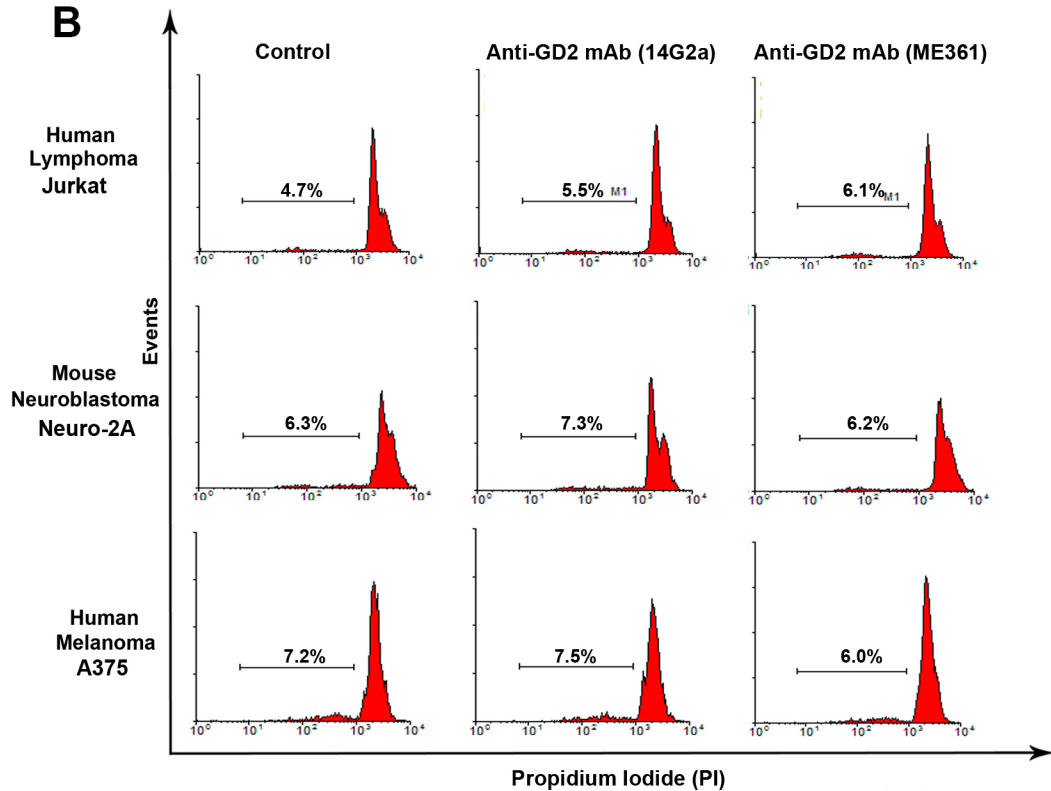

Supplement: Additional file 2 — The cytotoxic effects of anti-GD2 antibodies on GD2-negative tumor cell lines. Phase-contrast images of GD2-negative tumor cell lines Jurkat, Neuro-2a, and A375 after 24 h of incubation with or without anti-GD2 mAbs, 14G2a (5 μg/ml) and ME361 (5 μg/ml) are shown in (A). Analysis of DNA fragmentation (PI assay; see Methods) of GD2-negative tumor cell lines Jurkat, Neuro-2a, and A375 treated with GD2 mAbs 14G2a (5 μg/ml) and ME361 (5 μg/ml) is shown in (B). In (A), bar scale: 50 μm. In (B), percentages of the cells with fragmented DNA in hypodiploid peaks are shown for each histogram. [file 1471-2407-14-295-S2.pdf]

**A**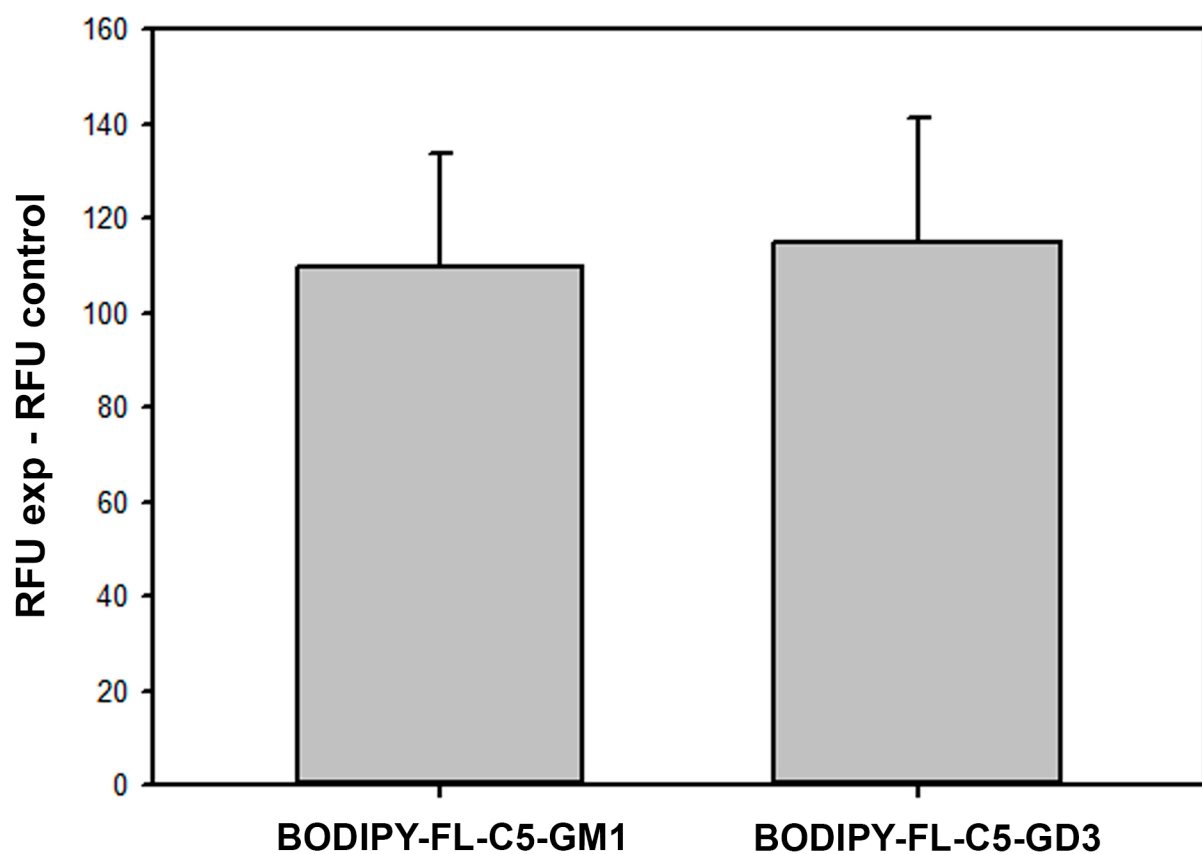**B**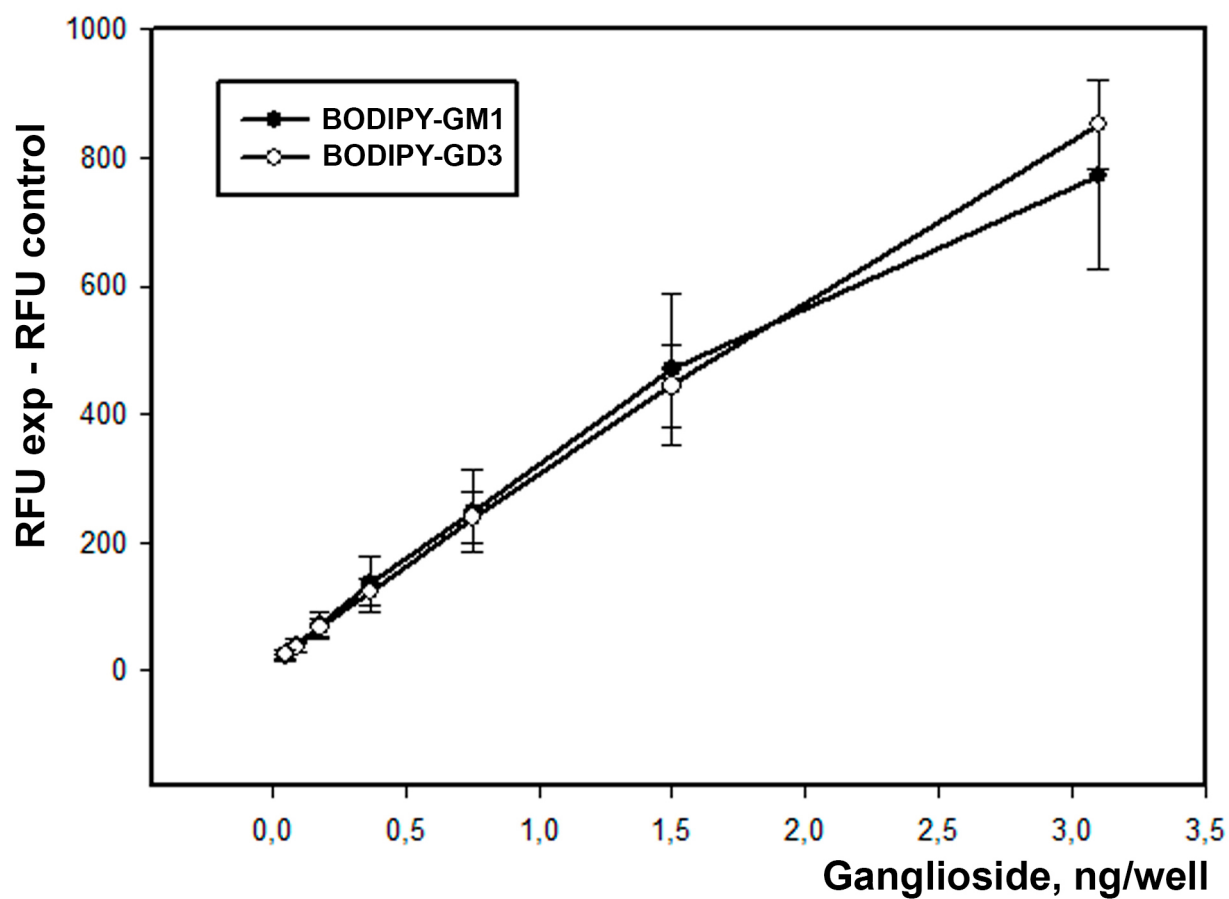

Supplement: Additional file 3 — Quantitative analysis of the gangliosides adsorbed on the ELISA plates. (A) The RFU (relative fluorescence units) level of fluorescent-labeled gangliosides BODIPY-FL-C5-GM1 and BODIPY-FL-C5-GD3 bound to the well before TMB reaction development in ELISA experiments is shown. Mean ± S.E. of nine separate experiments is shown. The RFU level was measured at 490 nm. The amount of BODIPY-FL-C5-GM1 bound to the well = 0.29 ± 0.04 ng, BODIPY-FL-C5-GD3 = 0.34 ± 0.05 ng which was measured using calibration curve. Statistical analysis was performed using Student’s t-test, there was not a statistically significant difference between BODIPY-FL-C5-GD3 and BODIPY-FL-C5-GM1groups (P = 0.765). (B) Calibration curve of fluorescent-labeled gangliosides BODIPY-FL-C5-GM1 and BODIPY-FL-C5-GD3 is shown, Linear regression: RFU BODIPY-FL-C5-GD3 = 20.726 + (271.329 × Amount of ganglioside per well), RFU BODIPY-FL-C5-GM1 = 36.396 + (248.714 × Amount of ganglioside per well, RFU - relative fluorescence units). Titration points are shown as Mean ± S.E of nine experiments. Statistical analysis was performed using Mann–Whitney rank sum test, there was no statistically significant difference between BODIPY-FL-C5-GD3 and BODIPY-FL-C5-GM1 (P = 0.911; not significant). [file 1471-2407-14-295-S3.pdf]
